# Camera Movement Impacts on Mu‐Wave Activity During Action Observation in Adults With Autism Spectrum Disorders Without Intellectual Disabilities

**DOI:** 10.1002/aur.70012

**Published:** 2025-02-27

**Authors:** Veronica Nisticò, Renata del Giudice, Francesca Serio, Giovanni Boido, Gianmarco Ingrosso, Francesco Lombardi, Claudio Sanguineti, Valeria Casula, Adelaide Baccara, Elia Chiudinelli, Francesca Vairano, Federica Maria Panzeri, Mauro Giori, Paolo Maria Inghilleri di Villadauro, Raffaella Faggioli, Orsola Gambini, Tomaso Subini, Benedetta Demartini

**Affiliations:** ^1^ Dipartimento di Scienze Della Salute Università Degli Studi di Milano Milano Italy; ^2^ “Aldo Ravelli” Research Center for Neurotechnology and Experimental Brain Therapeutics University of Milan Milan Italy; ^3^ Dipartimento di Psicologia Università Degli Studi di Milano‐Bicocca Milano Italy; ^4^ Unità di Psichiatria 52 Dipartimento Salute Mentale e Dipendenze, Presidio San Paolo, ASST Santi Paolo e Carlo Milano Italy; ^5^ Dipartimento di Beni Culturali e Ambientali Università Degli Studi di Milano Milano Italy

**Keywords:** autism spectrum disorders, EEG, Mirror neuron system, mu‐wave, perspective‐taking

## Abstract

The aim of this study was to investigate differences in mu‐wave modulation in individuals diagnosed with autism spectrum disorder (ASD) without intellectual disabilities compared to a group of neurotypical controls (NT). Thirty autistic individuals and 30 NT underwent an EEG recording while watching short videos depicting goal‐oriented action filmed from a fixed position, zooming in on the scene, and approaching the scene by means of a steadycam. Then, participants underwent a rating task to evaluate their subjective viewing experience. We found that steadycam videos elicited enhanced event‐related desynchronization (ERD), suggestive of enhanced neural activity, in the NT group, and a reduced ERD in the autistic group, compared to the other filming conditions. Autistic participants also showed difficulties in returning to baseline mu‐power levels after watching videos filmed from a fixed position. Finally, NT reported feeling more comfortable watching videos with movement, whereas autistic participants did not exhibit differences between conditions. We speculated that static, less naturalistic stimuli might impose higher and prolonged cognitive demands on autistic individuals. Understanding these differences might help develop tailored interventions to support perceptual, cognitive, and social processes of autistic people.

**Summary**

- Through electroencephalography (EEG), we investigated for the first time how the neuroatypical brain, particularly the so‐called mirror neuron system (believed to underpin our understanding of other people's perspectives), reacts to visual stimuli filmed with different techniques, one of which (Steadycam condition) is designed to emulate the real‐life context more than the others. We found that video filmed with the Steadycam enhanced the brain activity of neurotypical individuals and reduced it in people with ASD, compared to the other two filming techniques.

## 1 Introduction

Autism spectrum disorder (ASD) is a complex neurodevelopmental condition presenting with different physical, behavioral, and psychological features (such as anomalies in perception, integration, and processing of sensory stimuli often resulting in cognitive rigidity, resistance to change, inclination toward systematization, and social difficulties) (American Psychiatric Association 2022; Turner 1999; Rogers and Ozonoff 2005; Leekam et al. 2007; Tomchek and Dunn 2007). Despite extensive investigation, the neurophysiological underpinnings of ASD remain poorly understood. A predominant hypothesis relates to the activity of the brain's mu‐rhythm and the so‐called “mirror neuron system” (MNS), considered as an execution–imagination–observation matching system (Nishitani and Avikainen 2004; Williams 2006; Gallese 2006; Velikonja et al. 2019; Andreou and Skrimpa 2020; Yates and Hobson 2020). Mirror neurons (MN) are bimodal neurons located in the macaque's ventral premotor cortex, discharging both when a goal‐directed action is performed and when it is observed (Gallese et al. 1996). In humans, MN discharge during pantomiming and in response to communicative actions (Cattaneo and Rizzolatti 2009), corroborating the hypothesis that they might contribute to an embodied understanding of others' actions and goals, ultimately facilitating learning, imitation, and acquisition of motor abilities, skills, and behaviors. MN play a role in visual perspective‐taking, a fundamental social cognitive process acquired in infancy through sensory experiences and imitation (Noguchi et al. 2022), that enables one to understand the thoughts, intentions, and emotions of other individuals (Jeannerod and Anquetil 2008), and ultimately to experience empathy. Overall, MNS alterations can hinder the comprehension of others' mental states, with broader implications for social cognition.

The registration of the brain's mu‐rhythm through electroencephalography (EEG) was proposed as the ideal noninvasive technique to investigate the activity of the MNS (Oberman et al. 2005), as well as a potential therapeutic target for social cognition difficulties (Dastgheib et al. 2024). The mu‐rhythm (or mu‐wave, defined as the frequency band ranging 8–14 Hz topographically centered on the standard C3 and C4 positions over the sensorimotor cortex (Oberman et al. 2005; Muthukumaraswamy and Johnson 2004; Pineda 2005)), shows an attenuation known as event‐related desynchronization (ERD), usually followed by a physiological marked rebound known as event‐related synchronization (ERS) (Pfurtscheller et al. 1996, 1997; Pfurtscheller and Lopes da Silva 1999; Salmelin 1994) during: action execution (including passive or reflex movements) (Chatrian et al. 1959), observation of others' movements in movies (Gastaut and Bert 1954; Blume et al. 2015), and movement imagination (Pfurtscheller et al. 2006). Mu‐wave activity does not appear modulated by which body part is moving but by goal‐directed actions (Muthukumaraswamy and Johnson 2004). Moreover, mu‐wave suppression was found to be heightened when the stimulus depicted an actor facing the camera, enhancing perspective‐taking and emotional content perception, compared to stimuli with actors facing away from the camera (Kilner et al. 2006).

Mu‐wave studies in ASD have produced conflicting findings (Andreou and Skrimpa 2020; Lockhart et al. 2023). On the one hand, a diminished attenuation of the mu‐rhythm in individuals with ASD compared to neurotypical individuals (NT) was reported (Oberman et al. 2005), a finding that correlated with limited imitation skills (Bernier et al. 2007); observations revealing increased mu suppression in both NT and ASD children when observing familiar individuals performing grasping actions compared to unfamiliar individuals (Oberman et al. 2008) support the social relevance hypothesis (Kilner et al. 2006), suggesting that the reported MNS anomalies in ASD may stem from a lack of social relevance in the stimuli. On the other hand, Fan et al. (Fan et al. 2010) found that mu suppression during movement observation did not significantly differ between ASD and NT; importantly, studies employing more selective methodologies and participant criteria revealed more nuanced findings: for instance, mu alterations were detected in the upper mu‐band (10–13 Hz) but not in the lower mu‐band (8–10 Hz) (Dumas et al. 2014), and in ASD individuals with specific genetic profiles, such as likely gene‐disrupting mutations, but not in those without (Hudac et al. 2017).

These results prompted further inquiries about the methodology used in mu‐wave/MN studies (for a recent review see Kemmerer (2021)). One feature is the kind of stimuli shown to the participants, which are usually videos depicting a human being performing a grasping action: can more realistic and ecologically valid stimuli, achieved through improved filming techniques, influence mu suppression? Heimann et al. (2014) investigated NT's mu‐wave activation while observing videos showing goal‐oriented actions filmed using various techniques: from a fixed position, zooming in on the scene, or approaching the scene by means of a dolly or a steadycam (hence including the potential movement of the observer toward the actor). They found that videos filmed with a steadycam elicited a stronger ERD of the mu‐rhythm and were subjectively felt by NT to be more involving, emphasizing the importance of ecologically designed studies for exploring social cognition.

## 2 Aim of the Study

Previous studies investigating mu‐wave suppression in individuals with ASD are limited by having consistently employed video clips filmed from a fixed camera position. Here, firstly, we aimed to explore the impact of various filming techniques on the potential modulation of the mu‐wave in individuals with neuroatypical functioning; secondly, through a rating task, we aimed to understand the level of involvement experienced by the participants while observing the videos and to assess their perception of the ecological plausibility associated with the different types of camera movements employed in the recordings. Hence, we replicated the protocol designed by Heinmann and colleagues (Heimann et al. 2014) within a group of adults with ASD without intellectual disabilities compared to a group of NT.

## 3 Materials and Methods

### 3.1 Participants

Sample numerosity was determined through a priori power analysis G. Power 3.1 (Faul et al. 2009): given *α* = 0.05, Power (1‐β) = 0.80, and effect size = 0.20 (Cohen 1988), the required total sample size should have had a numerosity of at least *N* = 42 (Supporting Information). To accommodate potential dropouts and EEG registration failures, we set the sample size at *N* = 60. Thirty individuals with ASD were recruited from ASST Santi Paolo e Carlo, Presidio S. Paolo. Diagnosis followed DSM‐5 criteria and assessment with ADOS‐2 (Hus and Lord 2014), Autism Spectrum Quotient (AQ) (Baron‐Cohen et al. 2001), Ritvo Autism and Asperger Diagnostic Scale‐Revised (RAADS‐R) (Ritvo et al. 2011), Empathy Quotient (EQ) (Baron‐Cohen and Wheelwright 2004), and Sensory Perception Quotient‐Short Form (Tavassoli et al. 2014) (Supporting Information). Thirty NT, matched for biological sex and age (± 5 years), were recruited from the general population via word‐of‐mouth. Exclusion criteria were: (i) age < 18 years (ii) intellectual disabilities, that is, IQ < 75, measured via the Wechsler Adult Intelligence Scale‐Fourth Edition (WAIS‐IV) (Lang et al. 2015) administered to the ASD group participants during the diagnostic assessment and to NT before taking part in the study; (iii) inability to understand the instructions of the task; (iv) being left‐handed, assessed with the Edinburgh Handedness Inventory (Oldfield 1971); (v) history of seizures and/or neurosurgical intervention; (vi) presence of intracranial ferromagnetic material; (vii) being treated with psychopharmacological or neurological therapies (i.e., antidepressants, tranquilizers, antipsychotics, mood stabilizers, antiepileptics or antiparkinsonians); (viii) substance use disorder; and (ix) for NT only: scoring above the cut‐off at AQ (≥ 32), or at RAADS‐R (≥ 66), and having first‐degree relatives diagnosed with ASD, to minimize potential genetic or environmental influences (i.e., subthreshold autistic traits).

The study received approval from the Ethics Committee of Milano Area 1 (Registro Sperimentazioni 2021/ST/258, Protocol N0016813) and was conducted in accordance with the Declaration of Helsinki. All participants signed an informed consent form.

### 3.2 Procedure

The experiment, consisting of a 60 min EEG recording session followed by a 10 min rating task, took place during the daytime in a softly lit, sound‐attenuated room.

Participants sat on a comfortable chair, with eyes open, resting both their arms on their legs, in front of a computer screen placed on a table at 50 cm. A high‐density EEG cap was positioned as follows: 62 electrodes mounted on the scalp according to the International 10/20 System and referenced to FPz; four electrooculogram (EOG) recorded in the horizontal and vertical axes; two electrodes applied to the auricular lobes. Impedances were kept below 20 kΩ. The signal was acquired with a sampling rate of 512 Hz, recording in DC, with the software g. Recorder (G. Tec Medical Engineering GMBH, Austria). Stimuli were presented with E‐Prime 3.0 (Psychology Software Tools, Pittsburgh, PA).

Heinmann et al. (Faul et al. 2009) shared the stimuli implemented in their study. The videos, each lasting 3 s, featured an agent (50% a woman, 50% a man, unfamiliar to the participants) grasping from a table the following objects: a scotch tape, a battery, a plastic glass, an eraser, an 8 cm radius plastic ball, a pack of tissues, a marble, and an espresso cup. Videos were recorded from a fixed position, zooming in on the scene and approaching the scene with a steadycam to simulate the observer's approach. Camera movement started 260 cm from the object and, in cases of movement, ended 80 cm from the object; speed and height were consistent across conditions.

**FIGURE 1:**
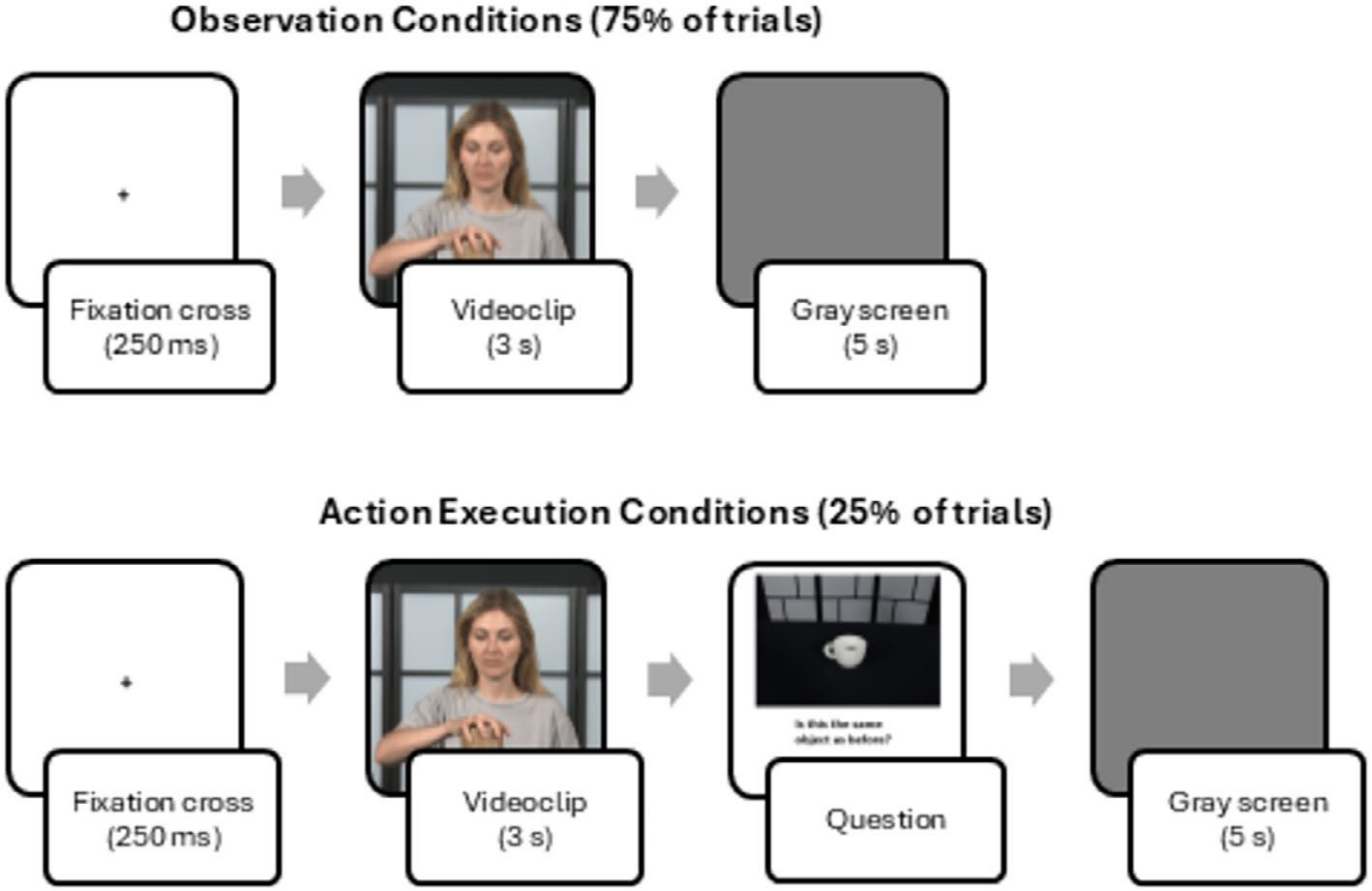
Experimental paradigm. In 75% of the trials, participants were presented with: (I) a fixation cross of 250 ms, (ii) a 3 s video depicting an actor grasping an object, (iii) a 5 s gray screen. EEG‐data were analyzed for these trials only. In the remaining 25% of the trials, participants were presented with: (I) a fixation cross of 250 ms, (ii) a 3 s video depicting an actor grasping an object, (iii) a picture of an object with the question “is this the object you saw before?”, to be replied to with the mouse, (iv) a 5 s gray screen. These data were analyzed as index of attention towards the task.

Figure 1 presents the entire experimental design. EEG was recorded during six blocks of about 10 min length each, interspersed with a short break. Each block consisted of 64 trials, beginning with a fixation cross of 250 ms and followed by a 3 s video (presented in random order). In 75% of the trials (consisting of 16 Still videos, 16 Steadycam video, and 16 Zoom video), after stimulus presentation, a gray screen was displayed for 5 s to guarantee the return of brain activity to baseline; participants were asked to blink only in the second half of the gray screen period to minimize movement artifacts in the resynchronization phase. In the remaining 25% of the trials (16 trials randomly chosen among the Still, Steadycam, and Zoom conditions), after stimulus presentation and before the gray screen, the photo of an object appeared, and participants were asked to tell whether the object was the same one they had just seen being grasped in the video displayed before. The answer had to be given by clicking on a mouse (positioned on the table in front of them) with their right index finger and then returning to the baseline position. EEG recordings for the action execution trials were not analyzed: instead, only the number of correct and incorrect answers was calculated and considered as an index of each participant's attention. Participants were instructed to remain still throughout the procedure, except when answering questions or during breaks between blocks. Two experimenters independently monitored participants' movements and reported no significant activity that could have compromised EEG recordings during the observation or rest conditions.

In the rating task, participants were shown 12 videos (3 Still, 3 Zoom, 3 Steadycam; Table 1) in a randomized order. After each video, participants were asked to reply on a Visual Analogue Scale from 0 (not at all) to 100 (completely) to the following six questions: (1) “How much did you feel involved in the scene?”; (2) “How much did you feel like the actor?”; (3) “How much did you feel as if you yourself would approach the scene?”; (4) “How comfortable did you feel watching the scene?”; (5) “How realistic did you find the camera movement?”; (6) “How much did you feel the camera movement resembled a person's movement when approaching the scene?” (Supporting Information for the Italian translation). Questions 3, 5, and 6 were not asked in the Still conditions. Questions 1 to 3 aimed to investigate participants' potential feeling of involvement with the observed scene; question 4 to explore how much participants were at ease with the different filming conditions; questions 5 and 6 to measure participants' estimation of the ecological plausibility of the different types of camera movements (Faul et al. 2009).

**TABLE 1:** Details of the video shown during the rating task.

Video camera	Sex of the actor	Object grasped
Still	Male	Espresso cup
Still	Male	Eraser
Still	Female	Plastic ball
Still	Female	Marble
Steadycam	Male	Scotch tape
Steadycam	Male	Plastic glass
Steadycam	Female	Tissues
Steadycam	Female	Battery
Zoom	Male	Plastic ball
Zoom	Male	Marble
Zoom	Female	Espresso cup
Zoom	Female	Eraser

### 3.3 EEG Preprocessing and Analysis

EEG data were preprocessed using BrainVision Analyzer 2.2 software (Brain Products, Gilching, Germany). Data were first downsampled to 256 Hz; then the EEG signal was bandpass filtered between 0.5 and 80 Hz and notch‐filtered to correct for the main electrical noise (European 50 Hz). Data were re‐referenced to auricular channels. An ocular correction, using the regression‐based approach from Gratton et al. (1983), was applied, and residual artifacts (such as muscular activity, or technical artifacts as steep changes in voltage) were removed by a semiautomatic procedure and visual inspection (Villasana 2024). Subsequently, the data were segmented following Heimann et al. (2014): 2000 ms before the trigger and 6000 ms after the trigger, totaling 8000 ms. Segments were then clustered into three conditions (Still, Steadycam, Zoom) based on marker information. After artifact, trials, and channel removals, the EEG data of three NT and one individual with ASD were removed from subsequent analysis. Hence, the final EEG analysis was run on a subsample of 27 NT and 29 participants with ASD; overall, we analyzed: in the neurotypical group, 2110 trials in the Still condition, 2101 in the Steadycam condition, and 2154 in the Zoom condition; within the autistic group, 2053 trials in the Still condition, 2071 in the Steadycam condition, and 2104 in the Zoom condition. No differences between groups were found in terms of the number of errors committed in the action execution conditions, the number of channels rejected and interpolated for each subject during data cleaning, and the number of trials considered for each condition after trials rejection (all *p* > 0.05, Table 2).

**TABLE 2:** Sociodemographic and psychometric information.

	NT, Mean (SD)	ASD, Mean (SD)	*χ* or U, (df)	*p*
Age	36.03 (14.27)	35.87 (13.336)	453 (60)	0.965
Biological Sex, M/F	14/16	14/16	0 (1)	1
BMI	23.87 (4.75)	23.77 (3.46)	405.5 (55)	0.606
WAIS‐IV Intelligence Quotient	114.14 (13.63)	121.1 (15.2)	482 (56)	0.138
WAIS‐IV Verbal Comprehension	117.69 (14.63)	126.5 (14.8)	527 (56)	**0.026**
WAIS‐IV Perceptual Reasoning	109.24 (11.00)	119.3 (15.3)	539 (56)	**0.015**
WAIS‐IV Working Memory	109.86 (15.30)	154.5 (249.5)	357.5 (56)	0.577
WAIS‐IV Processing Speed	103.79 (12.50)	106.6 (17.1)	449.5 (56)	0.34
ADOS‐2 Communication	NA	3.97 (1.691)	NA	NA
ADOS‐2 Reciprocal social interaction	NA	7.37 (2.498)	NA	NA
ADOS‐2 Imagination/Creativity	NA	1.53 (0.681)	NA	NA
ADOS‐2 Stereotyped behaviors and restricted interests	NA	1.2 (1.126)	NA	NA
ADOS‐2 Total Social Communication	NA	11.47 (4.01)	NA	NA
AQ Total Score	13.27 (5.66)	35.59 (6.76)	803 (57)	**< 0.001**
AQ Social skills	1.60 (1.99)	7.56 (2.45)	766 (57)	**< 0.001**
AQ Attention switching	3.60 (1.49)	8.52 (1.45)	800.5 (57)	**< 0.001**
AQ Attention to detail	3.27 (1.94)	7.04 (2.24)	724 (57)	**< 0.001**
AQ Communication	2.23 (1.90)	7.22 (2.47)	762.5 (57)	**< 0.001**
AQ Imagination	2.57 (1.56)	5.26 (1.74)	706 (57)	**< 0.001**
RAADS‐R Total Score	33.50 (18.29)	148.3 (37.0)	810 (57)	**< 0.001**
RAADS‐R Social Relatedness	16.47 (10.64)	72.52 (23.6)	780 (57)	**< 0.001**
RAADS‐R Circumscribed Interests	6.27 (4.45)	30.81 (7.62)	810 (57)	**< 0.001**
RAADS‐R Language	3.07 (2.98)	11.78 (4.52)	771.5 (57)	**< 0.001**
RAADS‐R Sensory‐motor	7.70 (6.12)	32.67 (13.1)	775 (57)	**< 0.001**
SPQ‐SF35 Total Score	61.40 (11.96)	48.85 (16.07)	3.34 (54)	**0.002**
SPQ‐SF35 Vision	12.23 (3)	9.04 (4.16)	3.33 (54)	**0.002**
SPQ‐SF35 Smell	16.33 (4.29)	15.19 (6.13)	0.82 (54)	0.419
SPQ‐SF35 Taste	5.77 (2.34)	4.54 (2.28)	1.98 (54)	0.053
SPQ‐SF35 Touch	16.1 (4.34)	11.65 (4.41)	3.8 (54)	**< 0.001**
SPQ‐SF35 Hearing	10.97 (2.04)	8.42 (2.27)	4.42 (54)	**< 0.001**
EQ	47.76 (10.97)	22.34 (10.81)	37 (52)	**< 0.001**
N° errors committed in the action execution conditions	5.47 (4.78)	5.8 (6.15)	437.5 (60)	0.835
N° channels rejected and interpolated for each subject	0.43 (1.72)	0.2 (0.92)	434.5 (60)	0.633
N° trials considered ‐ Total	235.74 (46.69)	214.76 (60.63)	321 (56)	0.247
N° trials considered ‐ Still	78.15 (16.1)	70.79 (15.65)	324 (56)	0.268
N° trials considered ‐ Steadycam	77.81 (15.55)	71.41 (20.03)	332.5 (56)	0.333
N° trials considered ‐ Zoom	79.78 (15.65)	72.55 (20.01)	326 (56)	0.283

*Note*: Bold values *p* < 0.05.

Abbreviations: ADOS‐2 = Autism Diagnostic Observation Schedule—2nd version; ASD = Autism Spectrum Disorders without intellectual disabilities group; AQ = Autism Quotient; BMI = Body Mass Index; df = degrees of freedom; F = Female; M = Male; NT = Neurotypical group; p = significance threshold (*α* = 0.05); RAADS‐R = Ritvo Autism Asperger Diagnostic Scale‐Revised; SD = Standard deviation; WAIS‐IV = Wechsler Adult Intelligence Scale—Fourth Edition.

EEG segments were exported and analyzed using custom‐built Matlab routines (MathWorks, Natick, MA) and the EEGLAB toolbox (Delorme and Makeig 2004). Event‐related potentials (ERPs) of each cluster were calculated for each condition within each group. Event‐related spectral perturbation (ERSP) computation was performed using the function *timef* with 3‐cycle complex Morlet wavelets with an increasing factor of 0.8 and a minimum frequency of 3 Hz. The average of the 500 ms of the gray screen before the fixation cross (i.e., [−1500–1000] ms) was chosen as the baseline for ERD/ERS calculation. Wavelet transformation was calculated separately for each participant in all 62 scalp channels for each condition. Data were analyzed concerning power changes for Rolandic upper mu‐rhythm frequency (10–13 Hz) (Pfurtscheller and Lopes da Silva 1999; Dumas et al. 2014; Pfurtscheller and Aranibar 1979; Pfurtscheller and Berghold 1989; Perry et al. 2010) in the following consecutive time windows: Onset (of the video and the ERD, 0–1000 ms); ERD, corresponding to the grasping act in the video (1000–3000 ms); ERS (3000–4000 ms); Return‐to‐baseline (4000–5000 ms). As in Heimann et al. (2014), we restricted our focus to a selected cluster of six electrodes in each hemisphere located around standard C3 and C4 sites, based on the maximum distribution of the mu‐frequency (Oberman et al. 2005; Muthukumaraswamy and Johnson 2004; Pineda 2005): electrodes FT7‐FC5‐FC3‐T7‐C5‐C3, “Left Hemisphere”, and electrodes FC4‐FC6‐FT8‐C4‐C6‐T8, “Right Hemisphere”. For this cluster, ERPs (for each subject and grand average) and mean ERSP were calculated within each condition.

### 3.4 Statistical Analysis

#### 3.4.1 Sociodemographic and Psychometric Data

Statistical analyses were performed in SPSS 28; *α* < 0.05 deemed significant; all tests were two‐tailed. The Kolmogorov–Smirnov test confirmed that all the continuous variables followed a normal distribution. Descriptive statistics (sociodemographic and clinical information) were calculated for both samples, and potential differences between groups were investigated either via t‐test for independent samples (continuous variables) or via χ‐squared (categorical variables).

#### 3.4.2 EEG

We implemented a 2×4×2×3 repeated measures (RM) ANOVA, with mean ERSP values as the dependent variable; Time‐Window (Onset, ERD, ERS, Return‐to‐baseline), Group (NT vs. ASD), Hemisphere (Right vs. Left), and Condition (Still vs. Steadycam vs. Zoom) as between‐ and within‐subject factors. Since the assumption of homogeneity of variance was not met (Mauchly's test *p* < 0.05), results are reported according to Greenhouse–Geisser correction. Differences between groups and conditions within each hemisphere and time window were investigated via post hoc Bonferroni comparisons.

#### 3.4.3 Rating Task

A series of linear mixed models were run, with Subject as the clustering variable (random intercept), the answers to each question (from 1 to 6 and their reaction times, separately) as the dependent variables, and Group (ASD vs. NT) as the independent variable, with respect to which the fixed effect was calculated; pairwise comparisons were run applying Bonferroni's correction.

## 4 Results

Groups were balanced for age, biological sex, BMI, and Total IQ (all *p* > 0.05). Table 2 shows sociodemographic and psychometric information.

### 4.1 EEG

**FIGURE 2:**
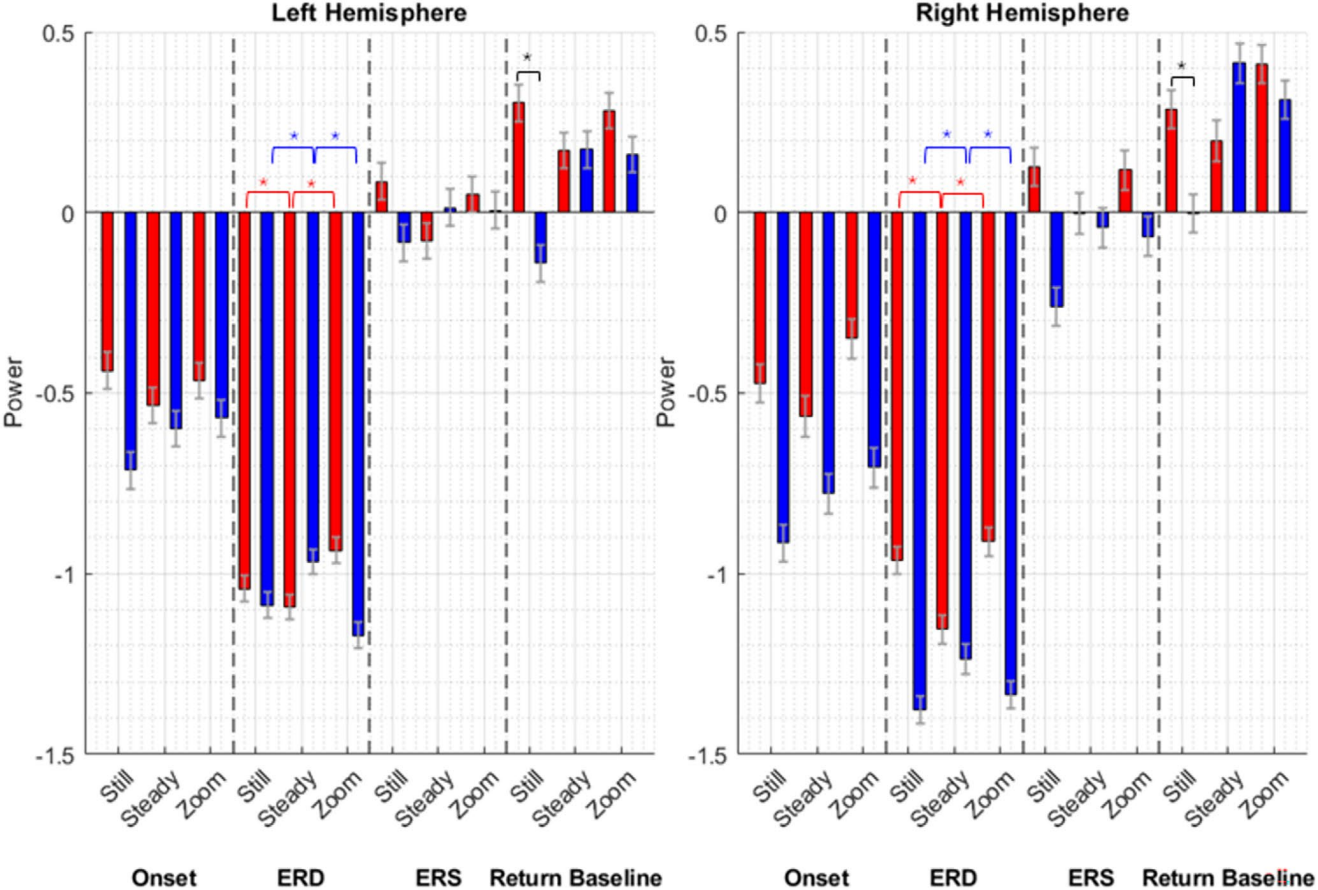
Results of the ANOVA comparing mu‐wave activity of autistic individuals (blu bars) and neurotypical individuals (red bars) across the four time‐windows. * = significant differences relevant to the main scope of our study: First, during the full ERD phase, NT participants showed more pronounced ERD in the Steadycam condition compared to Still and Zoom, while autistic participants showed reduced ERD in the Steadycam condition compared to Still and Zoom (all *p* < 0.005). Second, during the return to baseline, between‐group differences (represented in black) were observed in the Still condition for both hemispheres, where autistic participants showed below‐baseline power that was significantly less pronounced than NT. Full‐list significant differences are reported in the main text and in the Supporting Informations.

**FIGURE 3:**
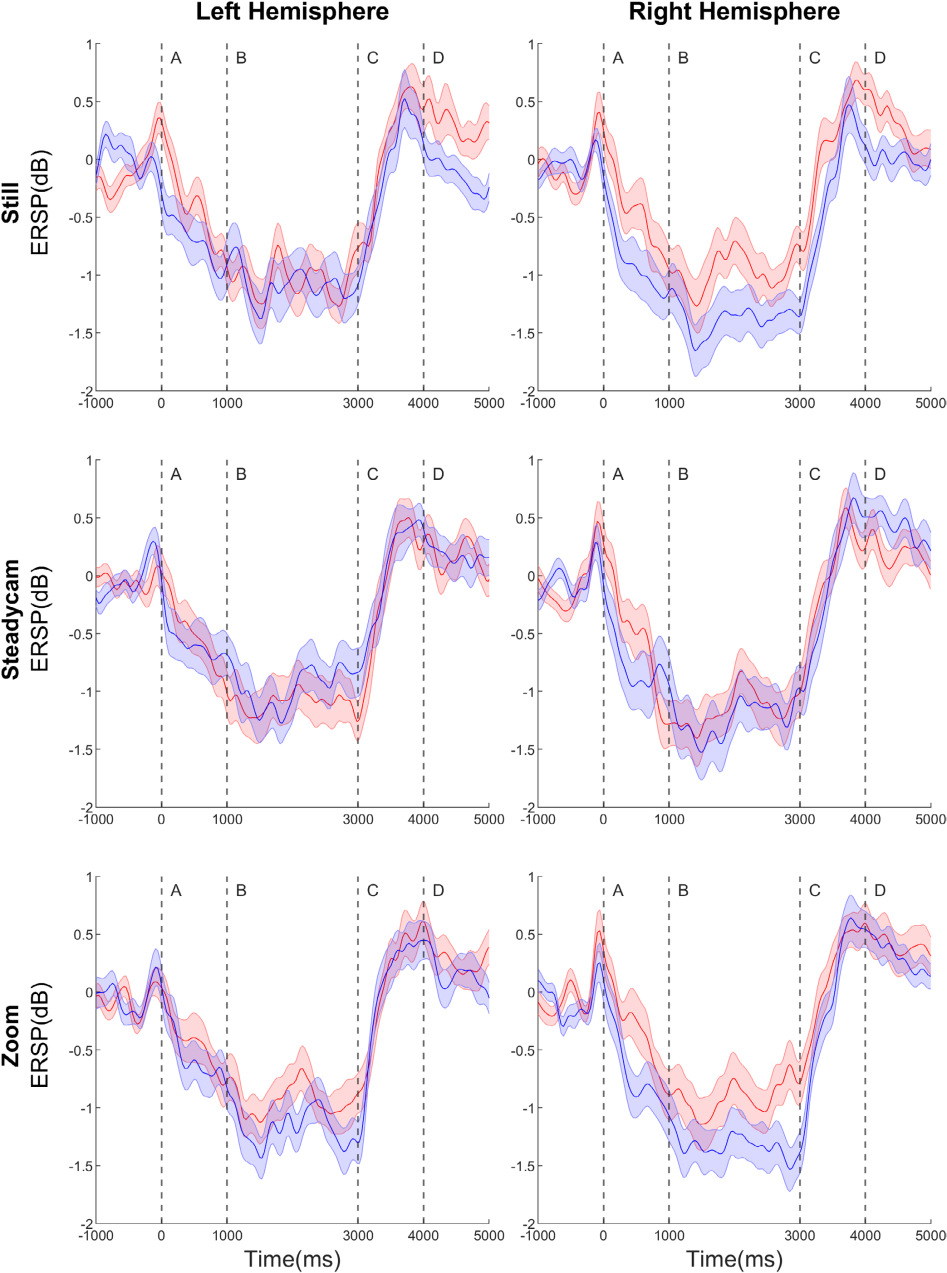
ERSP data. Comparison between groups (blue: autistic, red: neurotypical) of the event‐related spectral perturbation in the three conditions. All graphs represent the full time window, starting from ‐1500 ms and ending at 5500 ms. Vertical lines in each graph represent the significant time points separating the time windows of interest: A = Onset, B = ERD, C = ERS, D = Return to Baseline.

The RM‐ANOVA revealed a significant fourth‐level interaction between Camera, Hemisphere, Time‐window, and Group (F(5.87, 541.1) = 9.03, *p* < 0.001, εp^2^ = 0.09) (Figure 2, Table 3). Full statistical analyses and figures depicting ERPs, time‐frequency decomposition, and ERSP comparisons between groups for the Still, Steadycam, and Zoom conditions are provided in the Supporting Information. Figure 3 illustrates the average ERSP across the entire time window (−1500 ms to 5500 ms) for both groups, across the three conditions, and for both hemispheres.

**TABLE 3:** ANOVA results.

Hemisphere	Time window	Camera	NT	ASD	*p* Value Post‐hoc NT vs ASD	*p* Value Post‐hoc NT			*p* Value Post‐hoc ASD		
			Mean (St. Err.)	Mean (St. Err.)		vs Still	vs Steadycam	vs Zoom	vs Still	vs Steadycam	vs Zoom
Right	Onset	Still	−0.47 (0.05)	−0.92 (0.05)	**< 0.001**	1			1		
		Steadycam	−0.56 (0.06)	−0.78 (0.06)	**0.006**	**0.001**	1		**< 0.001**	1	
		Zoom	−0.35 (0.05)	−0.71 (0.05)	**< 0.001**	**< 0.001**	**< 0.001**	1	**< 0.001**	**0.013**	1
	ERD	Still	−0.96 (0.04)	−1.38 (0.04)	**< 0.001**	1			1		
		Steadycam	−1.15 (0.04)	−1.24 (0.04)	0.140	**< 0.001**	1		**< 0.001**	1	
		Zoom	−0.91 (0.04)	−1.34 (0.04)	**< 0.001**	**0.004**	**< 0.001**	1	**0.02**	**< 0.001**	1
	ERS	Still	0.13 (0.05)	−0.26 (0.05)	**< 0.001**	1			1		
		Steadycam	0.00 (0.06)	−0.04 (0.06)	0.615	**< 0.001**	1		**< 0.001**	1	
		Zoom	0.12 (0.05)	−0.07 (0.05)	**0.018**	1	**< 0.001**	1	**< 0.001**	0.997	1
	RetBaseline	Still	0.29 (0.05)	0.00 (0.05)	**< 0.001**	1			1		
		Steadycam	0.20 (0.06)	0.41 (0.06)	**0.007**	**0.002**	1		**< 0.001**	1	
		Zoom	0.41 (0.05)	0.31 (0.05)	0.211	**< 0.001**	**< 0.001**	1	**< 0.001**	**0.001**	1
Left	Onset	Still	−0.44 (0.05)	−0.71 (0.05)	**< 0.001**	1			1		
		Steadycam	−0.53 (0.05)	−0.60 (0.05)	0.342	**0.001**	1		**< 0.001**	1	
		Zoom	−0.46 (0.05)	−0.57 (0.05)	0.136	0.904	0.29	1	**< 0.001**	0.768	1
	ERD	Still	−1.04 (0.04)	−1.09 (0.04)	0.379	1			1		
		Steadycam	−1.09 (0.04)	−0.97 (0.04)	**0.012**	**0.02**	1		**< 0.001**	1	
		Zoom	−0.94 (0.04)	−1.17 (0.04)	**< 0.001**	< 0.001	**< 0.001**	1	**< 0.001**	**< 0.001**	1
	ERS	Still	0.09 (0.05)	−0.08 (0.05)	**0.020**	1			1		
		Steadycam	−0.08 (0.05)	0.01 (0.05)	0.192	**< 0.001**	1		**0.001**	1	
		Zoom	0.05 (0.05)	0.01 (0.05)	0.528	0.507	**< 0.001**	1	**0.001**	1	1
	RetBaseline	Still	0.30 (0.05)	−0.14 (0.05)	**< 0.001**	1			1		
		Steadycam	0.17 (0.05)	0.17 (0.05)	0.969	**< 0.001**	1		**< 0.001**	1	
		Zoom	0.28 (0.05)	0.16 (0.05)	0.086	1	**< 0.001**	1	**< 0.001**	1	1

*Note*: Bold values *p* < 0.05.

Abbreviations: ASD = Autism Spectrum Disorders without intellectual disabilities group; NT = Neurotypical group. Post hoc are Bonferroni corrected.

In the first time window (mu‐wave ERD Onset, 0–1000 ms), between‐group differences were observed in the right hemisphere across all conditions (Still, Steadycam, and Zoom) and in the left hemisphere for the Still condition, with autistic individuals showing more pronounced desynchronization compared to NT. Within‐group comparisons showed that NT participants exhibited greater ERD in the Steadycam condition compared to Still and Zoom in both hemispheres. In contrast, autistic participants demonstrated stronger ERD in the Still condition compared to Steadycam and Zoom in both hemispheres (all *p* < 0.03).

In the second time‐window (mu‐wave full ERD, 1000–3000 ms), which corresponded to the visualization of the actor's grasping action, between‐group differences emerged: (i) in the right hemisphere, autistic participants showed greater ERD than NT in the Still and Zoom conditions; (ii) in the left hemisphere, autistic participants exhibited less desynchronization than NT in the Steadycam condition but greater desynchronization in the Zoom condition. Within‐group analyses indicated that, in both hemispheres, NT participants showed more pronounced ERD in the Steadycam condition compared to Still and Zoom, while autistic participants showed reduced ERD in the Steadycam condition compared to Still and Zoom (all *p* < 0.005).

In the third time‐window (mu‐wave ERS, 3000–4000 ms, end of video), between‐group differences were observed: (i) in the right hemisphere, in the Still and Zoom conditions, autistic participants exhibited reduced ERS (below baseline) compared to NT participants; (ii) in the left hemisphere, for the Still condition, autistic participants also showed significantly reduced ERS. Within‐group analyses revealed that, in both hemispheres, NT participants displayed a reduced ERS in the Steadycam condition compared to Still and Zoom. Conversely, autistic participants showed a reduced ERS in the Still condition compared to the Steadycam and Zoom conditions (all *p* ≤ 0.001).

In the fourth time‐window (return‐to‐baseline, 4000–5000 ms), between‐group differences were observed in the Still condition for both hemispheres, where autistic participants showed below‐baseline power that was significantly less pronounced than NT; moreover, in the right hemisphere, autistic participants presented significantly greater power in the Steadycam condition compared to NT. Within‐group comparisons indicated that NT participants showed reduced power in the Steadycam condition compared to Still and Zoom in both hemispheres, while autistic participants demonstrated reduced power in the Still condition compared to Steadycam and Zoom (all *p* ≤ 0.001).

### 4.2 Rating Task

A significant main effect of Condition emerged across all questions (all *p* < 0.005, Supporting Information). In question 1, participants felt more involved in the Steadycam condition compared to both Zoom and Still conditions; the Zoom condition made participants feel more involved than the still condition. In question 2, participants felt more intensely like they were the actor in the steadycam condition and the zoom condition compared to the still condition, while no differences emerged between the zoom and steadycam conditions. In question 3, participants felt more intensely as if they were approaching the scene when presented with a video in the steadycam condition compared to the zoom condition. In question 4, participants felt more comfortable when watching videos in the zoom condition compared to the Still condition; no differences emerged between the steadycam condition and either the zoom or still conditions. Participants found the camera movement in the steadycam condition more realistic compared to the zoom condition (question 5), and they felt more intensely that the camera movement resembled a person's movement in the steadycam condition compared to the zoom condition (question 6).

With respect to the comparison between groups, a trend toward significance emerged in question 2 (F(1, 62) = 3.738, *p* = 0.058), with ASD participants feeling less intensely as if they were the actors of the scene compared to NT. At question 4, pairwise comparison showed that, within NT only, participants felt more comfortable when the camera was moving compared to the still condition (Steadycam: *p* = 0.013, Zoom: *p* < 0.001), while no differences emerged between conditions in the ASD group, as if they felt equally comfortable in every condition administered (Table 4).

**TABLE 4:** Rating task results.

		Still	Steadycam	Zoom
Question 1 Mean (SD)	Overall	6.23 (10.11)	20.14 (24.49)	15.88 (21.58)
	ASD	5.41 (9.87)	20.84 (25.60)	17.06 (24.44)
	NT	7.05 (10.32)	19.44 (23.42)	14.69 (18.31)
Question 2, Mean (SD)	Overall	5.68 (10.79)	10.81 (19.66)	9.09 (17.08)
	ASD	3.20 (8.89)	7.33 (17.57)	5.76 (15.37)
	NT	8.17 (11.93)	14.29 (21.06)	12.43 (18.09)
Question 3, Mean (SD)	Overall	NA	20.14 (24.49)	15.88 (21.58)
	ASD	NA	20.84 (25.60)	17.06 (24.44)
	NT	NA	19.44 (23.42)	14.69 (18.31)
Question 4, Mean (SD)	Overall	56.2 (35.8)	59.43 (33.14)	62.98 (31.39)
	ASD	54.44 (36.40)	53.89 (34.27)	58.45 (31.30)
	NT	57.95 (35.25)	64.96 (31.14)	67.52 (30.95)
Question 5, Mean (SD)	Overall	NA	20.14 (24.49)	15.88 (21.58)
	ASD	NA	20.84 (25.60)	17.06 (24.44)
	NT	NA	19.44 (23.42)	14.69 (18.31)
Question 6, Mean (SD)	Overall	NA	20.14 (24.49)	15.88 (21.58)
	ASD	NA	20.84 (25.60)	17.06 (24.44)
	NT	NA	19.44 (23.42)	14.69 (18.31)

Abbreviations: ASD = Autism Spectrum Disorders without intellectual disabilities group; NA = Not Applicable; NT = Neurotypical group; SD = Standard deviation.

## 5 Discussion

The main aim of this study was to investigate how camera movement in videos depicting grasping actions modulates the upper mu‐wave (10–13 Hz) activity in adults with Autism Spectrum Disorders without intellectual disabilities, compared to neurotypical controls. We explored three filming techniques (Still, Steadycam, and Zoom) and assessed: (i) participants' neural responses through EEG, and (ii) their engagement and perceptions through a rating task.

### 5.1 EEG

Both NT and autistic participants demonstrated mu‐wave event‐related desynchronization (ERD) during grasping observation across all conditions, confirming the activation of the action observation network. However, the patterns of mu‐wave modulation differed between groups and conditions, shedding light on potential neurophysiological differences.

In NT participants, the Steadycam condition elicited the strongest mu‐wave ERD, particularly in the right hemisphere. This finding replicates the results of Heimann and colleagues (Heimann et al. 2014), who found, in neurotypical individuals, a stronger ERD in response to videos filmed with a Steadycam compared to videos filmed from a fixed position and by two other artificial methods to simulate dynamic distance reduction (i.e., zoom and dolly). The authors speculated that reducing the distance between the observer and the observed agent, particularly using a filming technique that simulates the natural movement of a person toward the observer, would elicit more activity in the MNS during the observation of goal‐directed action. This is also in line with previous research (Aglioti et al. 2008; Calvo‐Merino et al. 2005, 2006) suggesting that motor cortex activation during action perception is weaker if the observed action is less familiar to the observer. In this case, the familiarity is perceptual, induced by the steadycam, leading to a stronger feeling of involvement in the scene. The fact that our findings are more pronounced in the right hemisphere in the Steadycam condition resonates with the established association of the right parietal cortex with processing emotional, social, and nonverbal cues: this includes interpreting body language, gestures, and tone of voice, as well as spatial abilities relevant to social contexts; ultimately, this lateralized activity likely contributes to perspective‐taking and theory of mind abilities (Keenan et al. 2005).

Autistic individuals exhibited consistently higher ERD than NT across most conditions, which adds to the contradictive findings observed in various mu‐wave studies in ASD (Andreou and Skrimpa 2020). Higher desynchronization has been proposed to indicate increased allocation of cognitive resources, such as attention, memory, and executive functions (Debnath et al. 2019), and ERD/ERS up to the beta frequency bands are thought to reflect cortical activation oscillations, representing readiness to process information (Tard et al. 2016; Yu et al. 2020; Zhou et al. 2023). Hence, the higher ERD observed in our ASD participants may reflect a greater cognitive effort experienced during grasping visualization compared to NT. Intriguingly, in the Steadycam condition, the ERD in ASD participants was notably reduced compared to the Still and Zoom conditions, in both hemispheres: this brings their response closer to that of NT participants, suggesting that naturalistic visual stimuli might reduce cognitive demand for ASD individuals.

Poststimulus resynchronization (ERS and Return‐to‐baseline phases) revealed additional group differences. NT participants exhibited robust resynchronization across conditions, while autistic participants showed slower recovery to baseline in the Still condition. We might speculate that static, less naturalistic stimuli may impose prolonged cognitive demands on autistic individuals; furthermore, residual desynchronization during the gray screen (post‐video baseline) highlights potential challenges in disengaging from cognitive processing, particularly when observing scenes with less dynamism, ultimately resulting in a having fewer cognitive resources available to perform other tasks.

### 5.2 Rating Task

At the behavioral level, our findings indicate that participants generally experienced greater engagement (Question 1) when viewing videos filmed using a steadycam compared to those filmed with zoom or still conditions. This suggests that employing a steadycam in filming creates a visual experience closely mirroring natural human vision during movement, leading to enhanced involvement, consistent with findings by Heimann et al. (Faul et al. 2009; Heimann et al. 2017, 2019; Stancák Jr and Pfurtscheller 1996). Participants with ASD, across the three conditions, felt less intensely as if they were the actor of the scene (Question 2), in comparison to NT (trend toward significance). This peculiarity might be explained by the difficulties in perspective‐taking, empathy, and theory of mind that individuals with ASD experience. Similarly, we observed that only the NT reported feeling more comfortable while watching the videos when the camera was in motion (both steadycam and zoom condition) compared to the still condition, and no differences were found between conditions in the ASD group, as if they felt equally comfortable in every condition administered, regardless the filming technique (Question 4). Do these results really reflect a difficulty in perspective‐taking by individuals with ASD, or might they encounter greater difficulties in grasping the differences of filming technique per se? The response to the three questions directly comparing the effects of the two movement conditions (Zoom vs. Steadycam) might help disambiguate these two aspects. Participants overall reported a stronger sensation of approaching the scene (Question 3), perceived the camera movement as more realistic (Question 5), and believed that the camera movement more effectively resembled a person's movement (Question 6) when presented with videos in the Steadycam condition. No significant differences were observed between ASD and NT groups in these aspects. Hence, the “third‐person” ability to recognize that the shooting technique is different and the Steadycam condition is more ecological and naturalistic (as it resembles human movements itself), does not seem affected in individuals with ASD; on the other hand, when it comes to “feel as if they were the actor”, hence a perspective‐taking aspect, people with ASD show a significantly higher difficult than NT, possibly reflecting the enhanced cognitive effort emerged from the EEG (Debnath et al. 2019).

### 5.3 Strengths, Limits, Conclusions

Most of the studies examining imitation, action observations, and their neurophysiological correlates are primarily conducted on children, teens, and young adults; here, we provided data conducted on a sample of 30 neuro‐atypical adults without intellectual disabilities, compared to a group of 30 NT matched for gender, age, BMI, and IQ; hence, this study contributes to our understanding of the unique characteristics of individuals with ASD and provides valuable insights into their perceptual and cognitive processes. Moreover, our ASD group included a matched number of males and females, despite the well‐known higher prevalence of ASD in the male population. Future perspectives include testing whether any sex‐related difference in our results exists, and if they might be influenced by the actor's sex.

We acknowledge as a limitation of our study the lack of assessment of potential associations with the severity of autistic traits and overall cognitive functioning, which have been linked to mu activity in previous literature. Since AQ, RAADS‐R, and WAIS‐IV scores were part of our exclusion criteria and directly influenced group selection, these measures are not independent of group membership. Adjusting our analyses for these variables or exploring their correlations with outcomes could introduce circularity and mislead the interpretation of group differences. A more comprehensive approach in future research could involve administering an independent measure of cognitive functioning and autistic traits, separate from exclusion criteria, to serve as a covariate in the statistical model (ANCOVA).

Second, in our study design, we focused specifically on mu‐wave differences during action observation, as previous literature suggests that movement execution is not impaired in autistic individuals (Oberman et al. 2005; Bernier et al. 2007). Additionally, we did not include an imitation condition, as participants demonstrated proficiency in grasping movements, and we did not expect expertise in this act to modulate mu‐wave activity differently than in NT (Bernier et al. 2007). Such conditions could be implemented in a future version of Heimann et al.'s paradigm.

Moreover, we did not systematically control for potential posterior alpha activity: since the mu frequency band overlaps with the posterior alpha frequency band (recorded from O1 and O2) and the generator for posterior alpha is stronger than that for mu, it is possible that our EEG recordings might have been affected by posterior alpha activity (Supporting Information) future studies could address this topic by incorporating conditions that allow differentiation between central and posterior alpha activity. An important limitation of our study, though shared with other EEG‐based studies, while mu‐waves are considered a mirror neuron index, challenges arise due to EEG's limited spatial resolution: although mu suppression during observed hand actions may selectively indicate MNS functioning on the sensorimotor cortex (Muthukumaraswamy and Johnson 2004; Pineda 2005), distinguishing it from broader action observation/execution network activity (such as the superior temporal sulcus or the inferior parietal cortex) requires higher‐resolution techniques, like combined fMRI and EEG (Pfurtscheller et al. 1997). Lastly, future studies should implement further analysis differentiating distinguishing the sample of ASD based on genetic profiling (Hudac et al. 2017) or potential comorbidities. which may provide further insight into individual variability in mu‐wave suppression.

In conclusion, our study revealed that, during the full‐ERD phase of watching grasping videos, the ecological nature of video recording (i.e., visual stimuli filmed with a Steadycam) elicits opposite mu‐wave modulation in the two groups: it enhances suppression in neurotypical individuals and reduces suppression in autistic individuals compared to videos filmed from a fixed position or with a zoomed‐in view. Furthermore, autistic participants showed slower recovery to baseline mu activity levels after viewing videos filmed from a fixed position. Overall, we speculated that static, less naturalistic stimuli might impose higher and prolonged cognitive demands on autistic individuals.

## Author Contributions

V.N.: conceptualization, data curation, formal analysis, investigation, methodology, visualization, writing – original draft. R.G.: data curation, formal analysis, investigation, methodology, software, supervision, writing review and editing. F.S., G.B., G.I., F.L., C.S., V.C., A.B., E.C., F.V., F.M.P.: data curation, investigation, writing – original draft. M.G., P.M.I.V., R.F., O.G., T.S., B.D.: conceptualization, funding acquisition, project administration, resources, supervision, writing‐review and editing.

## Conflicts of Interest

The authors declare no conflicts of interest.

## Supporting information


Data S1.

## Data Availability

The data that support the findings of this study are available on request from the corresponding author. The data are not publicly available due to privacy or ethical restrictions.
