# Supplementary material for: Camera Movement Impacts on Mu‐Wave Activity During Action Observation in Adults With Autism Spectrum Disorders Without Intellectual Disabilities
Source: Autism Res. 2025 Feb 27;18(4):774–87. doi: 10.1002/aur.70012 (PMC12015791; doi:10.1002/aur.70012)
Supplement: Supplementary file 1 — Data S1. [file AUR-18-774-s001.docx]

# **Supplementary Materials**

## **Power analysis**

F tests - ANOVA: Repeated measures, within-between interaction

Analysis: A priori: Compute required sample size

Input: Effect size f = 0.20

α err prob = 0.05

Power (1-β err prob) = 0.80

Number of groups = 2

Number of measurements = 3

Corr among rep measures = 0.5

Nonsphericity correction ε = 1

Output: Noncentrality parameter λ = 10.0800000

Critical F = 3.1107662

Numerator df = 2.0000000

Denominator df = 80.0000000

Total sample size = 42

Actual power = 0.8

## **Diagnostic questionnaires**

1. the Autism spectrum Quotient is a self-report validated questionnaire to measure the degree to which an adult without intellectual disabilities exhibits autistic traits; a Total Score ≥ 32 suggests the presence of ASD traits; five subscales were also calculated according to the authors’ instructions: Social skills, Attention switching, Attention to detail, Communication, Imagination.
2. the Ritvo Autism and Asperger Diagnostic Scale Revised (RAADS-R, Ritvo et al., 2011) is a self-report validated questionnaire usually implemented in clinical settings to support the diagnosis of ASD without intellectual disabilities; a Total Score > 65 suggests that the participant should be further assessed for ASD; four subscales were also calculated according to the authors’ instructions: Social Relatedness, Circumscribed Interests, Language, Sensory-Motor.
3. The Empathy Quotient (EQ – Baron-Cohen and Wheelwright, 2004) investigates levels of affective empathy and cognitive empathy in adults without intellectual disabilities. It is made up of 40 questions and, based on the total score (which varies from 0 to 80), the participant can be in one of the following four categories: “low empathy or empathy below average” (score 0-32), “empathy average” (score 33-53), “above average empathy” (score 53-63), “high empathy” (score 64-80).
4. Sensory Perception Quotient – ​​Short Form (SPQ-SF35 – Tavassoli et al., 2014) investigates hypersensitivity or hyposensitivity in the 5 sensory modalities (vision, hearing, smell, touch, taste) and has shown a good ability to discriminate between adults with ASD and neurotypical subjects. It consists of 35 items to be answered on a 4-point Likert scale (from 0 – “strongly agree” to 3 – “strongly disagree”.

## **Rating task – Questions in Italian as shown to the participants.**

1. How much did you feel involved in the scene?

Quanto ti sei sentito/a coinvolto/a nella scena?

1. How much did you feel like the actor?

Quanto ti sei sentito/a come se fossi l'attore?

1. How much did you feel as if you yourself would approach the scene?

Quanto ti sei sentito come se fossi tu stesso/a ad approcciarti alla scena?

1. How comfortable did you feel watching the scene?

Quanto ti sei sentito/a a tuo agio guardando la scena?

1. How realistic did you find the camera movement?

Quanto hai trovato realistico il movimento della videocamera?

1. How much did you feel the camera movement resembled a person’s movement when approaching the scene?

Quanto ti è sembrato che i movimenti della videocamera ricordassero i movimenti di una persona che approcciava la scena?

## **Rating task - Complete results**

Question 1: How much did you feel involved in the scene?

A significant main effect of Video-camera emerged (F(2, 664) = 54.166), p < 0.001); participants felt more involved in the Steady-cam condition with respect to both Zoom (p < 0.001) and Still (p = 0.006) conditions; moreover, the Zoom condition made participants feel more involved than the Still. No differences emerged between groups, or at the interaction effect between Group and Video-camera. With respect to reaction times, again a significant main effect of Video-camera emerged (F(2, 656) = 5.016), p = 0.007); participants showed a longer reaction time when the camera was moving with respect to the Still condition (Steady-cam: p = 0.039, Zoom: p = 0.01), while no differences emerged between the Zoom and Steady-cam conditions. There were no differences between groups, nor in the interaction effect between Group and Video-camera.

Question 2: How much did you feel like the actor?

A significant main effect of Video-camera emerged (F(2, 664) = 14.191), p < 0.001) participants felt more intensely like they were the actor in the Steady-cam condition (p < 0.001) and the Zoom condition (p = 0.002) with respect to the Still condition, while no differences emerged between the Zoom and Steady-cam conditions. A trend towards significance emerged with respect to the Group effect (F(1, 62) = 3.738, p = 0.058), with ASD participants feeling less intensely as if they were the actor of the scene, with respect to NT. No differences emerged in the interaction effect between Group and Video-camera, nor at the reaction times between groups and conditions.

Question 3: How much did you feel as if you yourself would approach the scene?

A significant main effect of Video-camera emerged (F(1, 422) = 8.523), p = 0.004); participants felt more intensely as if they were approaching the scene when presented with a video in the Steady-cam condition with respect to the Zoom condition. No differences emerged between groups, nor in the interaction effect between Group and Video-camera, nor at the reaction times between groups and conditions.

Question 4: How comfortable did you feel watching the scene?

A significant main effect of Video-camera emerged (F(2, 664) = 7.661), p = 0.001); participants felt more comfortable when watching video in the Zoom condition, with respect to the Still condition (p < 0.001); no differences emerged between the Steady-cam condition and either the Zoom and the Still condition. There was no significant main effect of group, but a trend towards significant in the interaction effect Group*Condition was found (F (2, 664) = 2.548, p = 0.079). Pairwise comparison showed that within the NT group only, participants felt more comfortable when the camera was moving with respect to the Still condition (Steady-cam: p = 0.013, Zoom: p < 0.001), while no differences emerged between the Zoom and Steady-cam condition; on the other hand, no differences were found between conditions in the ASD group, as if they felt equally comfortable in every condition administered. Analysing the reaction times, we found a significant main effect of Video-camera (F2, 656, p = 0.004), with participants replying faster in the Steady-cam condition with respect to both the Zoom (p = 0.009) and the Still (p = 0.013) condition. There was no difference between the Zoom and Still condition, nor between the two groups.

Question 5: How realistic did you find the camera movement?

Participants overall found more realistic the camera movement in the Steady-cam condition with respect to the Zoom condition (F(1, 422) = 8.532, p = 0.004). No differences emerged between groups, or at the interaction effect between Group and Video-camera, nor at the reaction times.

Question 6: How much did you feel the camera movement resembled a person’s movement when approaching the scene?

Participants overall felt more intensely that the camera movement resembled a person’s movement in the Steady-cam condition with respect to the Zoom condition (F(1, 422) = 8.523, p = 0.004). No differences emerged between groups, or at the interaction effect between Group and Video-camera, nor at the reaction times. For further details, see the table below.

|  | | **Still** | **Steady-cam** | **Zoom** |
| --- | --- | --- | --- | --- |
| Question 1_Value,  mean (SD) | Overall | 6.23  (10.11) | 20.14  (24.49) | 15.88  (21.58) |
|  | ASD | 5.41  (9.87) | 20.84  (25.60) | 17.06  (24.44) |
|  | NT | 7.05  (10.32) | 19.44  (23.42) | 14.69  (18.31) |
| Question 1_Reaction time,  mean (SD) | Overall | 3666.59  (7254.37) | 5137.20  (6787.22) | 5404.47  (7736.65) |
|  | ASD | 3249.27  (7614.20) | 5980.99  (8436.28) | 5532.97  (7922.15) |
|  | NT | 4083.92  (6882.35) | 4293.41  (4462.42) | 5275.97  (7577.65) |
| Question 2_Value,  mean (SD) | Overall | 5.68  (10.79) | 10.81  (19.66) | 9.09  (17.08) |
|  | ASD | 3.20  (8.89) | 7.33  (17.57) | 5.76  (15.37) |
|  | NT | 8.17  (11.93) | 14.29  (21.06) | 12.43  (18.09) |
| Question 2_Reaction time,  mean (SD) | Overall | 2468  (4723.2) | 2687.30  (4529.18) | 2565.07  (4032.14) |
|  | ASD | 1944.33  (4569.59) | 2687.23  (5346.60) | 2461.20  (4467.20) |
|  | NT | 2991.68  (4834.30) | 2687.38  (3551.51) | 2668.94  (3560.24) |
| Question 3_Value,  mean (SD) | Overall | NA | 20.14  (24.49) | 15.88  (21.58) |
|  | ASD | NA | 20.84  (25.60) | 17.06  (24.44) |
|  | NT | NA | 19.44  (23.42) | 14.69  (18.31) |
| Question 3_Reaction time,  mean (SD) | Overall | NA | 4863.82  (4624.63) | 5047.68  (5837.30) |
|  | ASD | NA | 4912.51  (4773.02) | 5043.73  (6277.82) |
|  | NT | NA | 4815.13  (4490.84) | 5051.64  (5387.33) |
| Question 4_Value,  mean (SD) | Overall | 56.2  (35.8) | 59.43  (33.14) | 62.98  (31.39) |
|  | ASD | 54.44  (36.40) | 53.89  (34.27) | 58.45  (31.30) |
|  | NT | 57.95  (35.25) | 64.96  (31.14) | 67.52  (30.95) |
| Question 4_Reaction time,  mean (SD) | Overall | 6886.01  (6075.91) | 5657.30  (4433.96) | 6944.75  (5210.96) |
|  | ASD | 7517.29  (6726.89) | 5875.24  (3823.93) | 7697.58  (5194.43) |
|  | NT | 6254.73  (5299.88) | 5439.37  (4976.65) | 6191.93  (5139.18) |
| Question 5_Value,  mean (SD) | Overall | NA | 20.14  (24.49) | 15.88  (21.58) |
|  | ASD | NA | 20.84  (25.60) | 17.06  (24.44) |
|  | NT | NA | 19.44  (23.42) | 14.69  (18.31) |
| Question 5_Reaction time,  mean (SD) | Overall | NA | 4863.82  (4624.63) | 5047.68  (5837.30) |
|  | ASD | NA | 4912.51  (4773.02) | 5043.73  (6277.82) |
|  | NT | NA | 4815.13  (4490.84) | 5051.64  (5387.33) |
| Question 6_Value,  mean (SD) | Overall | NA | 20.14  (24.49) | 15.88  (21.58) |
|  | ASD | NA | 20.84  (25.60) | 17.06  (24.44) |
|  | NT | NA | 19.44  (23.42) | 14.69  (18.31) |
| Question 6_Reaction time,  mean (SD) | Overall | NA | 4863.82  (4624.63) | 5047.68  (5837.30) |
|  | ASD | NA | 4912.51  (4773.02) | 5043.73  (6277.82) |
|  | NT | NA | 4815.13  (4490.84) | 5051.64  (5387.33) |

## **EEG: Repeated Measure ANOVA**

### *Code – SPSS Sintax*

GLM Still_R Still_L Steady_R Steady_L Zoom_R Zoom_L BY Time_window Group

/WSFACTOR=Camera 3 Polynomial Hemisphere 2 Polynomial

/METHOD=SSTYPE(3)

/EMMEANS=TABLES(Time_window*Group*Camera*Hemisphere) COMPARE(Time_window) ADJ(BONFERRONI)

/EMMEANS=TABLES(Time_window*Group*Camera*Hemisphere) COMPARE(Group) ADJ(BONFERRONI)

/EMMEANS=TABLES(Time_window*Group*Camera*Hemisphere) COMPARE(Camera) ADJ(BONFERRONI)

/EMMEANS=TABLES(Time_window*Group*Camera*Hemisphere) COMPARE(Hemisphere) ADJ(BONFERRONI)

/PRINT=ETASQ OPOWER

/CRITERIA=ALPHA(.05)

/WSDESIGN=Camera Hemisphere Camera*Hemisphere

/DESIGN=Time_window Group Time_window*Group.

### *Results*

| **Within-subjects effects** | | | | |  |
| --- | --- | --- | --- | --- | --- |
|  | Type III sum of squares | df | F | p value | ε_p_^2^ |
| Camera | 2.438 | 1.922 | 96.989 | **< 0.001** | 0.257 |
| Camera * Time window | 1.215 | 5.767 | 16.111 | **< 0.001** | 0.147 |
| Camera * Group | 6.639 | 1.922 | 264.147 | **< 0.001** | 0.485 |
| Camera * Time window * Group | 1.967 | 5.767 | 26.092 | **< 0.001** | 0.218 |
| Errore(Camera) | 7.037 | 560 |  |  |  |
| Hemisphere | 0.256 | 1 | 22.579 | **< 0.001** | 0.075 |
| Hemisphere * Time window | 3.073 | 3.000 | 90.218 | **< 0.001** | 0.492 |
| Hemisphere * Group | 1.410 | 1.000 | 124.172 | **< 0.001** | **0.307** |
| Hemisphere * Time window * Group | 2.216 | 3.000 | 65.066 | **< 0.001** | 0.411 |
| Error(Hemisphere) | 3.179 | 280 |  |  |  |
| Camera * Hemisphere | 0.354 | 1.958 | 30.105 | **< 0.001** | 0.097 |
| Camera * Hemisphere * Time window | 0.320 | 5.873 | 9.054 | **< 0.001** | 0.088 |
| Camera * Hemisphere * Group | 0.127 | 1.958 | 10.793 | **< 0.001** | 0.037 |
| Camera * Hemisphere * Time window * Group | 0.319 | 5.873 | 9.031 | **< 0.001** | 0.088 |
| Error(Camera*Hemisphere) | 3.296 | 548.114 |  |  |  |
|  | | | | |  |
| **Between-subjects effects** | | | | |  |
|  | Type III sum of squares | df | F | p value | ε_p_^2^ |
| Intercept | 221.951 | 1 | 517.157 | **< 0.001** | 0.649 |
| Time window | 514.826 | 3 | 399.857 | **< 0.001** | 0.811 |
| Group | 11.061 | 1 | 25.772 | **< 0.001** | 0.084 |
| Time window * Group | 0.888 | 3 | 0.689 | 0.559 | 0.007 |
| Error | 120.169 | 280 |  |  |  |

| **Estimated Marginal Means** | | | | | | | |
| --- | --- | --- | --- | --- | --- | --- | --- |
| Hemisphere | Time window | Group | Camera | Estimated Marginal Mean | Standard error | 95% C.I. Inf Lim | 95% C.I. Sup Lim |
| Right | Onset | NT | Still | -0.472 | 0.053 | -0.577 | -0.367 |
|  |  |  | Steady | -0.564 | 0.056 | -0.673 | -0.454 |
|  |  |  | Zoom | -0.350 | 0.054 | -0.457 | -0.243 |
|  |  | ASD | Still | -0.916 | 0.053 | -1.021 | -0.811 |
|  |  |  | Steady | -0.779 | 0.056 | -0.889 | -0.670 |
|  |  |  | Zoom | -0.707 | 0.054 | -0.815 | -0.600 |
|  | ERD | NT | Still | -0.964 | 0.038 | -1.039 | -0.889 |
|  |  |  | Steady | -1.155 | 0.040 | -1.232 | -1.077 |
|  |  |  | Zoom | -0.912 | 0.039 | -0.989 | -0.836 |
|  |  | ASD | Still | -1.378 | 0.038 | -1.453 | -1.303 |
|  |  |  | Steady | -1.237 | 0.040 | -1.315 | -1.159 |
|  |  |  | Zoom | -1.335 | 0.039 | -1.412 | -1.259 |
|  | ERS | NT | Still | 0.126 | 0.053 | 0.021 | 0.231 |
|  |  |  | Steady | -0.002 | 0.056 | -0.111 | 0.107 |
|  |  |  | Zoom | 0.117 | 0.054 | 0.010 | 0.224 |
|  |  | ASD | Still | -0.261 | 0.053 | -0.366 | -0.156 |
|  |  |  | Steady | -0.042 | 0.056 | -0.151 | 0.068 |
|  |  |  | Zoom | -0.066 | 0.054 | -0.173 | 0.041 |
|  | Return to baseline | NT | Still | 0.287 | 0.053 | 0.182 | 0.392 |
|  |  |  | Steady | 0.198 | 0.056 | 0.089 | 0.307 |
|  |  |  | Zoom | 0.410 | 0.054 | 0.303 | 0.517 |
|  |  | ASD | Still | -0.004 | 0.053 | -0.110 | 0.101 |
|  |  |  | Steady | 0.413 | 0.056 | 0.304 | 0.522 |
|  |  |  | Zoom | 0.313 | 0.054 | 0.206 | 0.421 |
| Left | Onset | NT | Still | -0.438 | 0.051 | -0.539 | -0.338 |
|  |  |  | Steady | -0.534 | 0.050 | -0.631 | -0.436 |
|  |  |  | Zoom | -0.465 | 0.050 | -0.563 | -0.366 |
|  |  | ASD | Still | -0.714 | 0.051 | -0.815 | -0.614 |
|  |  |  | Steady | -0.600 | 0.050 | -0.698 | -0.503 |
|  |  |  | Zoom | -0.570 | 0.050 | -0.669 | -0.472 |
|  | ERD | NT | Still | -1.042 | 0.036 | -1.114 | -0.970 |
|  |  |  | Steady | -1.093 | 0.035 | -1.163 | -1.024 |
|  |  |  | Zoom | -0.936 | 0.036 | -1.006 | -0.866 |
|  |  | ASD | Still | -1.087 | 0.036 | -1.159 | -1.016 |
|  |  |  | Steady | -0.967 | 0.035 | -1.037 | -0.898 |
|  |  |  | Zoom | -1.171 | 0.036 | -1.241 | -1.101 |
|  | ERS | NT | Still | 0.086 | 0.051 | -0.015 | 0.186 |
|  |  |  | Steady | -0.078 | 0.050 | -0.175 | 0.020 |
|  |  |  | Zoom | 0.051 | 0.050 | -0.047 | 0.149 |
|  |  | ASD | Still | -0.083 | 0.051 | -0.184 | 0.017 |
|  |  |  | Steady | 0.014 | 0.050 | -0.083 | 0.112 |
|  |  |  | Zoom | 0.006 | 0.050 | -0.092 | 0.105 |
|  | Return to baseline | NT | Still | 0.304 | 0.051 | 0.203 | 0.404 |
|  |  |  | Steady | 0.172 | 0.050 | 0.074 | 0.269 |
|  |  |  | Zoom | 0.282 | 0.050 | 0.184 | 0.381 |
|  |  | ASD | Still | -0.141 | 0.051 | -0.241 | -0.040 |
|  |  |  | Steady | 0.174 | 0.050 | 0.077 | 0.272 |
|  |  |  | Zoom | 0.160 | 0.050 | 0.062 | 0.259 |

| **Bonferroni comparisons based on group** | | | | | | | | | |
| --- | --- | --- | --- | --- | --- | --- | --- | --- | --- |
| Hemisphere | Time window | Camera | (I) Group | (J) Group | Mean difference (2-1) | Standard error | p value | 95% C.I. Inf Lim | 95% C.I. Sup Lim |
| Right | Onset | Still | NT | ASD | .444 | 0.076 | 0.000 | 0.296 | 0.593 |
|  |  | Steady | NT | ASD | .216 | 0.079 | 0.006 | 0.061 | 0.370 |
|  |  | Zoom | NT | ASD | .357 | 0.077 | 0.000 | 0.206 | 0.509 |
|  | ERD | Still | NT | ASD | .414 | 0.054 | 0.000 | 0.308 | 0.520 |
|  |  | Steady | NT | ASD | 0.083 | 0.056 | 0.140 | -0.027 | 0.193 |
|  |  | Zoom | NT | ASD | .423 | 0.055 | 0.000 | 0.315 | 0.531 |
|  | ERS | Still | NT | ASD | .387 | 0.076 | 0.000 | 0.238 | 0.536 |
|  |  | Steady | NT | ASD | 0.040 | 0.079 | 0.615 | -0.115 | 0.194 |
|  |  | Zoom | NT | ASD | .183 | 0.077 | 0.018 | 0.031 | 0.335 |
|  | Return to baseline | Still | NT | ASD | .292 | 0.076 | 0.000 | 0.143 | 0.440 |
|  |  | Steady | NT | ASD | -.215 | 0.079 | 0.007 | -0.370 | -0.061 |
|  |  | Zoom | NT | ASD | 0.097 | 0.077 | 0.211 | -0.055 | 0.248 |
| Left | Onset | Still | NT | ASD | .276 | 0.072 | 0.000 | 0.134 | 0.418 |
|  |  | Steady | NT | ASD | 0.067 | 0.070 | 0.342 | -0.071 | 0.205 |
|  |  | Zoom | NT | ASD | 0.106 | 0.071 | 0.136 | -0.033 | 0.244 |
|  | ERD | Still | NT | ASD | 0.045 | 0.051 | 0.379 | -0.056 | 0.147 |
|  |  | Steady | NT | ASD | -.126 | 0.050 | 0.012 | -0.224 | -0.028 |
|  |  | Zoom | NT | ASD | .235 | 0.050 | 0.000 | 0.136 | 0.334 |
|  | ERS | Still | NT | ASD | .169 | 0.072 | 0.020 | 0.027 | 0.311 |
|  |  | Steady | NT | ASD | -0.092 | 0.070 | 0.192 | -0.230 | 0.046 |
|  |  | Zoom | NT | ASD | 0.045 | 0.071 | 0.528 | -0.094 | 0.184 |
|  | Return to baseline | Still | NT | ASD | .445 | 0.072 | 0.000 | 0.303 | 0.587 |
|  |  | Steady | NT | ASD | -0.003 | 0.070 | 0.969 | -0.141 | 0.135 |
|  |  | Zoom | NT | ASD | 0.122 | 0.071 | 0.086 | -0.017 | 0.261 |

| **Bonferroni comparisons based on camera movements** | | | | | | | | | |
| --- | --- | --- | --- | --- | --- | --- | --- | --- | --- |
| Hemisphere | Time window | Group | Camera 1 | Camera 2 | Mean difference (2-1) | Standard error | p value | 95% C.I. Inf Lim | 95% C.I. Sup Lim |
| Right | Onset | NT | Still | Steady | .092 | 0.025 | 0.001 | 0.030 | 0.153 |
|  |  |  | Still | Zoom | -.122 | 0.023 | 0.000 | -0.176 | -0.067 |
|  |  |  | Steady | Zoom | -.214 | 0.025 | 0.000 | -0.274 | -0.154 |
|  |  | ASD | Still | Steady | -.137 | 0.025 | 0.000 | -0.198 | -0.076 |
|  |  |  | Still | Zoom | -.209 | 0.023 | 0.000 | -0.264 | -0.154 |
|  |  |  | Steady | Zoom | -.072 | 0.025 | 0.013 | -0.132 | -0.012 |
|  | ERD | NT | Still | Steady | .190 | 0.018 | 0.000 | 0.146 | 0.234 |
|  |  |  | Still | Zoom | -.052 | 0.016 | 0.004 | -0.091 | -0.013 |
|  |  |  | Steady | Zoom | -.242 | 0.018 | 0.000 | -0.285 | -0.200 |
|  |  | ASD | Still | Steady | -.141 | 0.018 | 0.000 | -0.184 | -0.097 |
|  |  |  | Still | Zoom | -.043 | 0.016 | 0.025 | -0.082 | -0.004 |
|  |  |  | Steady | Zoom | .098 | 0.018 | 0.000 | 0.055 | 0.141 |
|  | ERS | NT | Still | Steady | .128 | 0.025 | 0.000 | 0.067 | 0.190 |
|  |  |  | Still | Zoom | 0.009 | 0.023 | 1.000 | -0.045 | 0.064 |
|  |  |  | Steady | Zoom | -.119 | 0.025 | 0.000 | -0.179 | -0.059 |
|  |  | ASD | Still | Steady | -.220 | 0.025 | 0.000 | -0.281 | -0.158 |
|  |  |  | Still | Zoom | -.195 | 0.023 | 0.000 | -0.250 | -0.141 |
|  |  |  | Steady | Zoom | 0.024 | 0.025 | 0.997 | -0.036 | 0.084 |
|  | Return to baseline | NT | Still | Steady | .089 | 0.025 | 0.002 | 0.028 | 0.151 |
|  |  |  | Still | Zoom | -.123 | 0.023 | 0.000 | -0.177 | -0.068 |
|  |  |  | Steady | Zoom | -.212 | 0.025 | 0.000 | -0.272 | -0.152 |
|  |  | ASD | Still | Steady | -.417 | 0.025 | 0.000 | -0.479 | -0.356 |
|  |  |  | Still | Zoom | -.318 | 0.023 | 0.000 | -0.372 | -0.263 |
|  |  |  | Steady | Zoom | .100 | 0.025 | 0.000 | 0.040 | 0.160 |
| Left | Onset | NT | Still | Steady | .095 | 0.026 | 0.001 | 0.032 | 0.158 |
|  |  |  | Still | Zoom | 0.026 | 0.025 | 0.904 | -0.035 | 0.087 |
|  |  |  | Steady | Zoom | -.069 | 0.026 | 0.029 | -0.132 | -0.005 |
|  |  | ASD | Still | Steady | -.114 | 0.026 | 0.000 | -0.178 | -0.051 |
|  |  |  | Still | Zoom | -.144 | 0.025 | 0.000 | -0.205 | -0.083 |
|  |  |  | Steady | Zoom | -0.030 | 0.026 | 0.768 | -0.093 | 0.033 |
|  | ERD | NT | Still | Steady | .051 | 0.019 | 0.020 | 0.006 | 0.097 |
|  |  |  | Still | Zoom | -.106 | 0.018 | 0.000 | -0.149 | -0.062 |
|  |  |  | Steady | Zoom | -.157 | 0.019 | 0.000 | -0.202 | -0.112 |
|  |  | ASD | Still | Steady | -.120 | 0.019 | 0.000 | -0.165 | -0.075 |
|  |  |  | Still | Zoom | .084 | 0.018 | 0.000 | 0.040 | 0.127 |
|  |  |  | Steady | Zoom | .204 | 0.019 | 0.000 | 0.159 | 0.249 |
|  | ERS | NT | Still | Steady | .163 | 0.026 | 0.000 | 0.100 | 0.227 |
|  |  |  | Still | Zoom | 0.035 | 0.025 | 0.507 | -0.026 | 0.096 |
|  |  |  | Steady | Zoom | -.128 | 0.026 | 0.000 | -0.192 | -0.065 |
|  |  | ASD | Still | Steady | -.098 | 0.026 | 0.001 | -0.161 | -0.034 |
|  |  |  | Still | Zoom | -.090 | 0.025 | 0.001 | -0.151 | -0.029 |
|  |  |  | Steady | Zoom | 0.008 | 0.026 | 1.000 | -0.056 | 0.071 |
|  | Return to baseline | NT | Still | Steady | .132 | 0.026 | 0.000 | 0.069 | 0.196 |
|  |  |  | Still | Zoom | 0.022 | 0.025 | 1.000 | -0.040 | 0.083 |
|  |  |  | Steady | Zoom | -.111 | 0.026 | 0.000 | -0.174 | -0.047 |
|  |  | ASD | Still | Steady | -.315 | 0.026 | 0.000 | -0.379 | -0.252 |
|  |  |  | Still | Zoom | -.301 | 0.025 | 0.000 | -0.362 | -0.240 |
|  |  |  | Steady | Zoom | 0.014 | 0.026 | 1.000 | -0.050 | 0.077 |

## **Event-related potentials and Results pertaining to the full alpha band (8-14 Hz)**


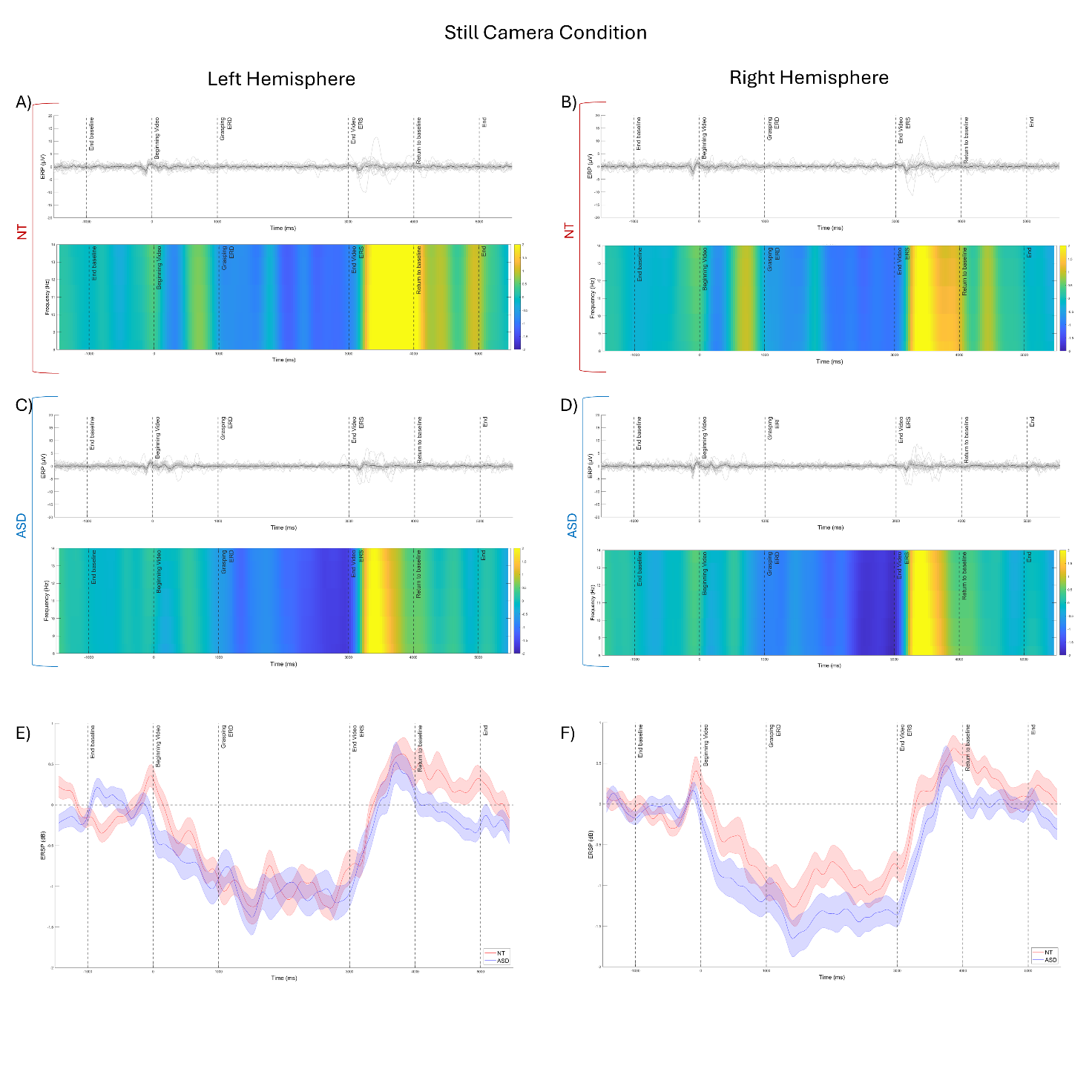


**EEG data related to the Still Camera condition. (A, B, C, D)** Event-related potential (above) and Time-Frequency decomposition (below) for, respectively: neurotypical group, left hemisphere; neurotypical group, right hemisphere; group of participants with ASD, left hemisphere; group of participants with ASD, right hemisphere; **(E, D)** Comparison between groups (red: neurotypical; blue: ASD) of the event-related spectral perturbation (average ERSP with shaded error bars), for the left and right hemisphere respectively. All graphs represent the full time-window, starting from -1500ms and ending at 5500ms. Vertical lines in each graph represent the significant time-points separating the time-windows of interest. Abbreviations: ASD = Autism Spectrum Disorder group, NT = Neurotypical group.


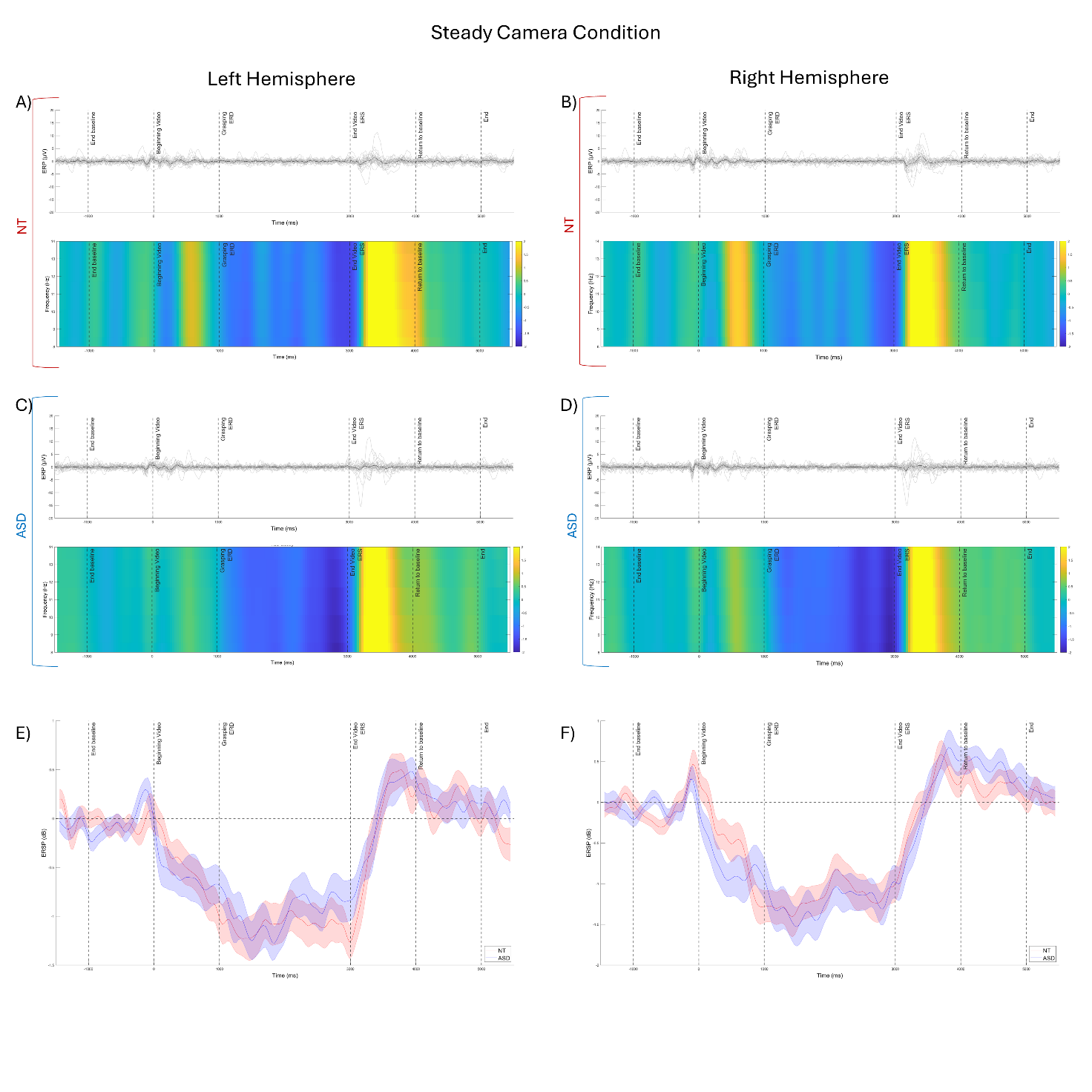


**EEG data related to the Steady Camera condition. (A, B, C, D)** Event-related potential (above) and Time-Frequency decomposition (below) for, respectively: neurotypical group, left hemisphere; neurotypical group, right hemisphere; group of participants with ASD, left hemisphere; group of participants with ASD, right hemisphere; **(E, D)** Comparison between groups (red: neurotypical; blue: ASD) of the event-related spectral perturbation (average ERSP with shaded error bars), for the left and right hemisphere respectively. All graphs represent the full time-window, starting from -1500ms and ending at 5500ms. Vertical lines in each graph represent the significant time-points separating the time-windows of interest. Abbreviations: ASD = Autism Spectrum Disorder group, NT = Neurotypical group.


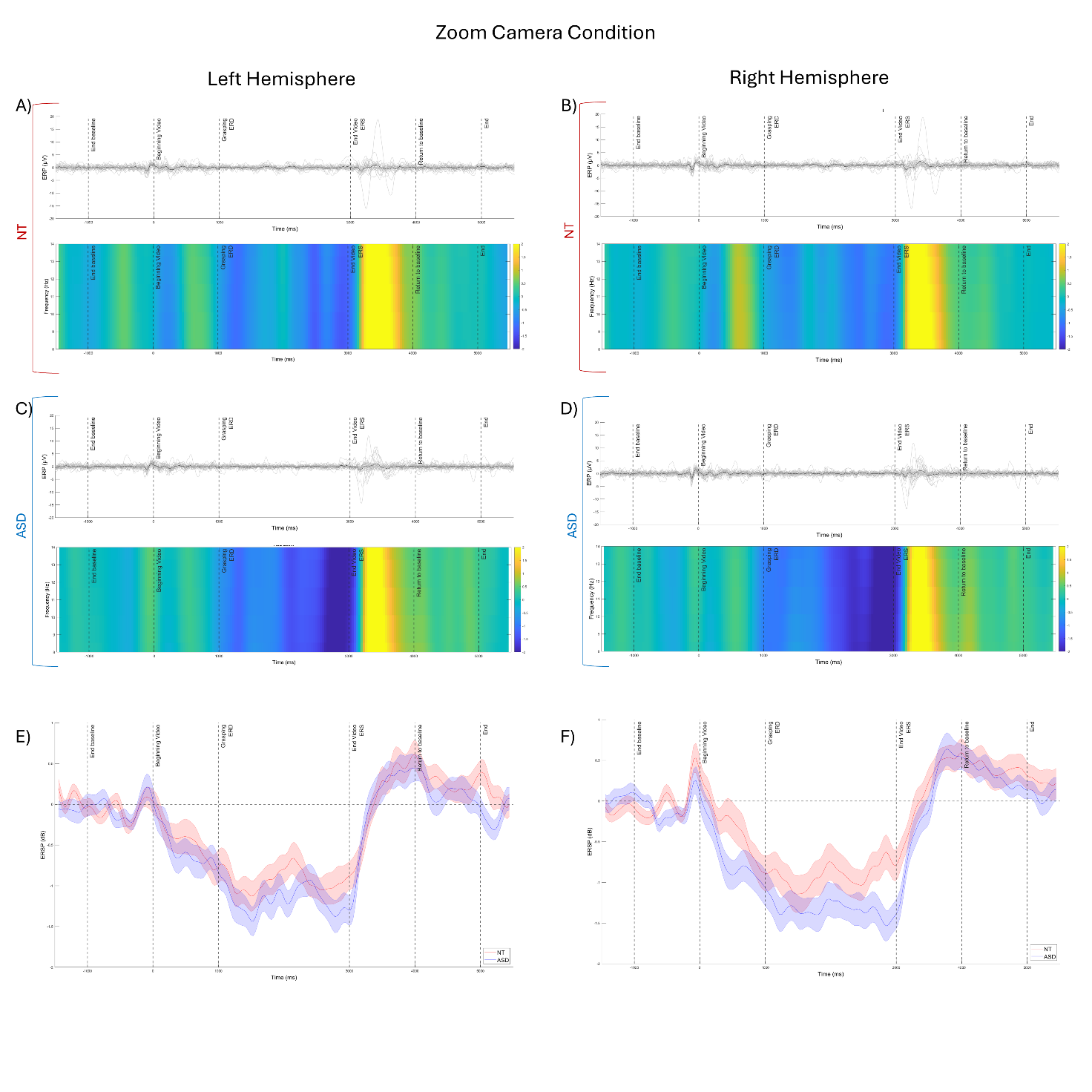


**EEG data related to the Zoom Camera condition. (A, B, C, D)** Event-related potential (above) and Time-Frequency decomposition (below) for, respectively: neurotypical group, left hemisphere; neurotypical group, right hemisphere; group of participants with ASD, left hemisphere; group of participants with ASD, right hemisphere; **(E, D)** Comparison between groups (red: neurotypical; blue: ASD) of the event-related spectral perturbation (average ERSP with shaded error bars), for the left and right hemisphere respectively. All graphs represent the full time-window, starting from -1500ms and ending at 5500ms. Vertical lines in each graph represent the significant time-points separating the time-windows of interest. Abbreviations: ASD = Autism Spectrum Disorder group, NT = Neurotypical group.

## **Supplementary Analysis for posterior alpha activity**

We run a RM ANOVA comparing mu wave activity in the occipital region (O1 and O2 channel) between Group, Conditions (Still, Steadycam, Zoom) and time window: we found a significant second-level interaction effect Group*Condition*Time window, with time windows significantly different between in each other in both groups, Group showing significant differences in most of the conditions, and some significant differences between conditions within each group, as shown in the table below. These results might open to the discussion of visual perception differences (intended at a lower level than the Observation-Action matching system) between neurotypical and neuroatypical subjects, which is beyond the scope of the present study, but deserve attention in further research,

| Time window | Camera | | Groups | | | | Mean difference | | St. Err. | | p (Bonferroni corrected) | | 95% C.I.) | |
| --- | --- | --- | --- | --- | --- | --- | --- | --- | --- | --- | --- | --- | --- | --- |
| Beginning | Still | | NT | | ASD | | .715^*^ | | 0.134 | | 0.000 | | [0.451; 0.978] | |
|  | Steady | | NT | | ASD | | .373^*^ | | 0.147 | | 0.011 | | [0.085; 0.661] | |
|  | Zoom | | NT | | ASD | | .579^*^ | | 0.145 | | 0.000 | | [0.294; 0.863] | |
| ERD | Still | | NT | | ASD | | .801^*^ | | 0.095 | | 0.000 | | [0.613; 0.989] | |
|  | Steady | | NT | | ASD | | .377^*^ | | 0.104 | | 0.000 | | [0.171; 0.582] | |
|  | Zoom | | NT | | ASD | | .635^*^ | | 0.103 | | 0.000 | | [0.432; 0.838] | |
| ERS | Still | | NT | | ASD | | .548^*^ | | 0.134 | | 0.000 | | [0.285; 0.812] | |
|  | Steady | | NT | | ASD | | .459^*^ | | 0.147 | | 0.002 | | [0.170; 0.747] | |
|  | Zoom | | NT | | ASD | | .328^*^ | | 0.145 | | 0.024 | | [0.044; 0.613] | |
| RetBaseline | Still | | NT | | ASD | | .656^*^ | | 0.134 | | 0.000 | | [0.392; 0.919] | |
|  | Steady | | NT | | ASD | | 0.184 | | 0.147 | | 0.210 | | [-0.104; -0.472] | |
|  | Zoom | | NT | | ASD | | .360^*^ | | 0.145 | | 0.013 | | [0.075; 0.644] | |
| Time_window | | **Group** | | **Camera conditions** | | | | **Mean difference** | | **St. Err.** | | **p (Bonferroni corrected)** | | **95% C.I.)** |
| Beginning | | NT | | Still | | Steady | | .183^*^ | | 0.034 | | 0.000 | | [0.101; 0.265] |
|  |  |  |  | Still | | Zoom | | -.079^*^ | | 0.032 | | 0.040 | | [-0.155; -0.003] |
|  |  |  |  | Steady | | Zoom | | -.262^*^ | | 0.028 | | 0.000 | | [-1.328; -1.195] |
|  |  | ASD | | Still | | Steady | | -.159^*^ | | 0.034 | | 0.000 | | [-0.241; -0.077] |
|  |  |  |  | Still | | Zoom | | -.215^*^ | | 0.032 | | 0.000 | | [-1.291; -1.139] |
|  |  |  |  | Steady | | Zoom | | -0.056 | | 0.028 | | 0.130 | | [-0.123; -0.010] |
| ERD | | NT | | Still | | Steady | | .330^*^ | | 0.024 | | 0.000 | | [-3.271; -3.388] |
|  |  |  |  | Still | | Zoom | | .092^*^ | | 0.023 | | 0.000 | | [-1.038; -1.147] |
|  |  |  |  | Steady | | Zoom | | -.237^*^ | | 0.020 | | 0.000 | | [-1.285; -1.190] |
|  |  | ASD | | Still | | Steady | | -.094^*^ | | 0.024 | | 0.000 | | [-0.153; -0.036] |
|  |  |  |  | Still | | Zoom | | -.073^*^ | | 0.023 | | 0.004 | | [-0.127; -0.018] |
|  |  |  |  | Steady | | Zoom | | 0.021 | | 0.020 | | 0.837 | | [-0.026; -0.069] |
| ERS | | NT | | Still | | Steady | | .144^*^ | | 0.034 | | 0.000 | | [-2.062; -2.226] |
|  |  |  |  | Still | | Zoom | | -0.060 | | 0.032 | | 0.184 | | [-0.136; -0.017] |
|  |  |  |  | Steady | | Zoom | | -.204^*^ | | 0.028 | | 0.000 | | [-1.270; -1.137] |
|  |  | ASD | | Still | | Steady | | 0.055 | | 0.034 | | 0.324 | | [-1.027; -1.137] |
|  |  |  |  | Still | | Zoom | | -.279^*^ | | 0.032 | | 0.000 | | [-2.356; -2.203] |
|  |  |  |  | Steady | | Zoom | | -.334^*^ | | 0.028 | | 0.000 | | [-2.401; -2.268] |
| RetBaseline | | NT | | Still | | Steady | | .259^*^ | | 0.034 | | 0.000 | | [-3.177; -3.341] |
|  |  |  |  | Still | | Zoom | | -0.014 | | 0.032 | | 1.000 | | [-0.090; -0.063] |
|  |  |  |  | Steady | | Zoom | | -.273^*^ | | 0.028 | | 0.000 | | [-2.339; -2.206] |
|  |  | ASD | | Still | | Steady | | -.213^*^ | | 0.034 | | 0.000 | | [-1.295; -1.131] |
|  |  |  |  | Still | | Zoom | | -.310^*^ | | 0.032 | | 0.000 | | [-2.386; -2.234] |
|  |  |  |  | Steady | | Zoom | | -.097^*^ | | 0.028 | | 0.002 | | [-0.163; -0.030] |
